# Breaking Glucose Transporter 1/Pyruvate Kinase M2 Glycolytic Loop Is Required for Cantharidin Inhibition of Metastasis in Highly Metastatic Breast Cancer

**DOI:** 10.3389/fphar.2019.00590

**Published:** 2019-05-24

**Authors:** Yanhong Pan, Qian Zheng, Wenting Ni, Zhonghong Wei, Suyun Yu, Qi Jia, Meng Wang, Aiyun Wang, Wenxing Chen, Yin Lu

**Affiliations:** ^1^Jiangsu Key Laboratory for Pharmacology and Safety Evaluation of Chinese Materia Medica, School of Pharmacy, Nanjing University of Chinese Medicine, Nanjing, China; ^2^Jiangsu Collaborative Innovation Center of Traditional Chinese Medicine (TCM) Prevention and Treatment of Tumor, Nanjing, China

**Keywords:** cantharidin, breast cancer, metastasis, aerobic glycolysis, pyruvate kinase M2, glucose transporter 1

## Abstract

Aerobic glycolysis plays a decisive role in cancer growth. However, its role in cancer metastasis was rarely understood. Cantharidin a natural compound from an arthropod insect cantharis exerts potent anticancer activity. Here we found cantharidin possesses significant anti-metastatic activity on breast cancer dependent on inhibition of aerobic glycolysis. Cantharidin indicates significant inhibition on migration and invasion of breast cancer cells, angiogenesis *in vitro*, and inhibits breast cancer cells metastasizing to liver and lung *in vivo*. Subsequent results revealed that cantharidin decreases the extracellular acidification rates (ECAR) but increases the oxygen consumption rates (OCR) in high metastatic cells, leading to suppression of aerobic glycolysis. This was considered to be due to inhibiting the activity of pyruvate kinase (PK) and further blocking pyruvate kinase M2 (PKM2) translocation in nucleus. Fructose-1,6-bisphosphate (FBP) and L-cysteine can significantly reverse cantharidin inhibition on breast cancer cell migration, invasion, and PKM2 translocation. Furthermore, glucose transporter 1 (GLUT1) forming a metabolic loop with PKM2 is downregulated, as well as epidermal growth factor receptor (EGFR), the regulator of the glycolytic loop. Totally, cantharidin inhibits the PKM2 nuclear translocation and breaks GLUT1/PKM2 glycolytic loop, resulting in aerobic glycolysis transformation to oxidation and subsequent reversing the metastases in breast cancer. Based on inhibiting multi signals mediated aerobic glycolysis, cantharidin could be prospectively used for prevention of metastasis in breast cancer patients.

## Introduction

According to the latest global cancer statistics in 2018, the incidence of breast cancer still following the lung cancer is on the second place and its 6.6% mortality lies first among the cancers of the female (Bray et al., 2018). The breast cancer is also one of the easiest metastasis carcinoma in the clinic. Over 90% of dead patients with breast cancer were confirmed to be due to metastasis (Gupta and Massagué, 2006). At present, although many new therapies such as cancer vaccine (Romero et al., 2016), tumor treating fields (Pless and Weinberg, 2011) and the immunotherapy (Sanmamed and Chen, 2018) become more prevalent for cancer metastasis, the chemotherapy is still the most important way. Specially, screening natural compounds for chemotherapy of metastasis and elucidating its molecular mechanism are also attractive and considerably concerned.

Cantharidin, a sesquiterpenoid bioactive component, is derived from beetles with high toxicity in the family of *Meloidae*. More than 1,500 beetle species in *Meloidae* family can secrete cantharidin (Abtahi et al., 2012). The *Mylabris cichorii Linnaeus* and *Mylabris phaleratus Pallas* are the most common blister beetles used in traditional Chinese medicine (Wu et al., 2018). Their dried insect bodies were used for more than 2,000 years due to their efficacy in activating blood and removing stasis, treating warts and molluscum through topical administration. And its action against molluscum is due to its high toxicity following the Chinese medicine theory of “fight poison with poison”, leading to subsequent discovery of other activities. In 1980, cantharidin was firstly demonstrated with the anti-cancer activity (Chen et al., 1980). In the past 40 years, the potential of cantharidin has been further explored, and its molecular target to cancer was primarily focused on serine/threonine protein phosphatases 1 and 2A (PP1 and PP2A) (Li and Casida, 1992). Following the extensive research, the critical molecular pathways by which cantharidin induces cancer growth inhibition and cell death were found to be more complicated. Cantharidin can induce cell apoptosis and inhibit the conversion of LC3-I to LC3-II and autophagosome formation by suppressing the expression of Beclin-1 (Li et al., 2017). Cantharidin triggers G2/M arrest and apoptosis by activating the mitochondrial caspase cascade (Huang et al., 2011). Moreover, cantharidin has been shown to increase the level of Bax, inhibit the level of Bcl-2 and survivin expression (Zhang et al., 2005), subsequently resulting in apoptotic cell death (Bonness et al., 2006). Particularly, cantharidin inhibits cancer cell migration and invasion *via* activating the IKKα/IκBα/NF-κB pathway by inhibiting PP2A activity (Zhou et al., 2018) or suppressing the MMPs (Ji et al., 2015; Shen et al., 2015), suggesting it may be a potential anti-metastatic compound. However, its anti-metastatic mechanism is far less understood.

As cantharidin is from *Mylabris* containing many active ingredients which have been demonstrated to possess the anti-cancer activity, we firstly made a comprehensive analysis on the *Mylabris* for assessment of its possible molecular mechanism based on the multidatabases. The enrichment analysis results indicated the most correlative pathway between the targets and active ingredients in *Mylabris* was focused on the metabolic pathways. Cancer is a metabolic disease and cancer cells prefer to produce energy by aerobic glycolysis to meet their rapid proliferation. Thus, we hypothesized that cantharidin inhibits metastasis possibly dependent on the aerobic glycolysis. Meanwhile, aerobic glycolysis almost controlling the whole process of cancer growth was regulated by many kinases including hexokinase (HK), PK, and phosphofructokinase (PFK). However, their role in cancer metastasis is not completely clear and needs to be further investigated. Here, we found cantharidin inhibiting metastasis is tightly related to inhibition of aerobic glycolysis. And further data indicated that cantharidin can not only block the nuclear localization of PKM2 one isoform of PK, but also inhibit GLUT1-mediated glycolytic signaling, thereby resulting in significant inhibition on the metastasis of breast cancer.

## Materials and Methods

### Chemicals and Bioreagents

Cantharidin (purity≥98%) was purchased from Chengdu Must Bio-Technology Co., Ltd (Chengdu, China). Modified Eagle Medium (DMEM), fetal bovine serum (FBS), trypsin-EDTA, and penicillin/streptomycin were from Gibco (Gibco, Grand Island, NY, USA). Primary antibodies to PKM2, GLUT1, and MCT1 were obtained from Abcam (Abcam, Cambridge, UK). Antibody against EGFR, PIN1, importin α5, and LDHA were purchased from CST (Cell Signaling Technology, Danvers, MA, USA), MCT4 from Proteintech, and β-actin and PKM from ABclonal (ABclonal, Woburn, MA, USA). D-(+)-Glucose solution, sodium pyruvate solution, and L-glutamine were provided by Sigma-Aldrich (Sigma-Aldrich, St. Louis, MO, USA). Other standard substances were obtained from Yuanye Biotechnology Co., Ltd (Shanghai, China). Glycolysis Stress Test Kit and Cell Mito Stress Test Kit were purchased from Seahorse Biosciences (Seahorse Biosciences, North Billerica, MA, USA).

### Cell Lines and Cell Culture

The human breast cancer cell line MDA-MB-231 (with highly metastatic property) and MCF-7 (with lowly metastatic property) from American type culture collection (ATCC) were cultured in DMEM with 10% FBS, and grown in a humidiﬁed-atmosphere incubator with 5% CO_2_ at 37°C.

### Wound Healing Assay

This assay was performed as previously described (Zhu et al., 2015). Briefly, 1.5×10^6^ cells/well breast cancer cells were piped into 6-well plates and grown into fully confluent monolayer culture. Mitomycin C (10 μg/ml) was used for inhibition of cell proliferation for 1 h. Then the confluent monolayer cells were scraped by a sterile pipette tip for wound, and the basal medium was replaced by the fresh medium containing cantharidin (0.1, 0.5, 1, 2 μM) or dimethyl sulfoxide (DMSO). The cell migration photos were taken by the ZEISS microscope, and the wound closure was calculated by Image J 1.5.

### Transwell Migration and Invasion Assay

The Corning Transwell system was used for the cell migration and invasion. A transwell chamber coated with or without 1–2 mg/ml Matrigel was used for measuring the cell migration and invasion, respectively. Cancer cells (4×10^5^ cells/transwell) were seeded into the upper chamber with serum-free medium and incubated with cantharidin (0, 0.1, 0.5, 1, and 2 μM), and 800-μl medium containing 30% FBS was piped into the lower chamber. After 24-h incubation at 37°C with 5% CO_2_, the medium was piped out of the upper chamber and the non-migrating or non-invading cells were removed by a cotton swab. The remaining cells were fixed by 4% paraformaldehyde, stained with 1% crystal violet solution, and washed with PBS. The number of invaded and migrated cells was counted per field of view at 200× magnification.

### Tube Formation Assay

The thawed Matrigel was mixed softly with complete medium containing 5×10^4^ of HUVEC by an equal volume ratio. Then it was treated with conditioned medium containing DMSO or different concentrations of cantharidin for 24 h. Finally, the photograph was taken by a ZEISS microscope and the number of completely formed tubes was counted using Image J1.5.

### Rat Aortic Ring Assay

According to the method described in previous study (Chen et al., 2011), this assay was performed with a modification. Firstly, thoracic aortas from Sprague–Dawley male rats were cut into 1-mm-wide rings, and ﬂushed with DMEM containing 10% FBS. Then rings were immediately placed into a 48-well plate containing Matrigel: DMEM/F12 complete medium (1:1, v/v) (400 μl per well) and incubated the plate at 37°C till the solution coagulated, followed with adding 200 μl DMEM/F12 complete medium containing DMSO or various concentrations of cantharidin into each well. During the 7-day treatment, tiny vascular vessels sprouting from each ring were carefully observed and photographed by a ZEISS microscope at 40× magnification.

### Oxygen Consumption Rates and Extracellular Acidification Rates Analysis

The oxygen consumption rates (OCR) and the extracellular acidification rates (ECAR) were measured using the Seahorse XFe24 Extracellular Flux Analyzer (Seahorse Biosciences, USA) according to the manufacturer’s protocol; 1×10^4^ cancer cells were seeded into a 24-well plate followed with overnight incubation, then treated with various concentrations of cantharidin for 24 h. After the cells were washed with Seahorse assay medium, 10 μM oligomycin, 2.5 μM FCCP, and 5 μM rotenone/antimycin A were automatically and successively injected to measure the OCR. To determine the ECAR, 100 mM glucose, 10 μM oligomycin, and 500 mM 2-deoxy-glucose (2-DG) were added into the solution. The OCR and ECAR values were calculated by a normalization to the cell number.

### Metabolites Analysis by HPLC-MS

The metabolites in glycolysis were determined by high performance liquid chromatography-mass spectrometry (HPLC-MS). Cell lysates were extracted in extraction buffer containing methanol: water mix in a 1:1 ratio with 5 mM ammonium acetate in the cold room for 15 min, then scraped and centrifuged at 17,000*g* for 10 min. Liquid chromatography (Prominence LC-20A) equipped with a tandem quadrupole mass spectrometry (QTRAP^®^5500, AB Sciex) and a 4.6 mm × 150 mm StableBond column (ZORBAX SB-AQ 5µm; Agilent) was used for metabolic flux analysis. The chromatography condition was: start with 98% solution B (0.1% formic acid in H_2_O) and 2% solution A (0.1% formic acid in methanol), gradient down to 80% solution B in 4 min, and back to 98% solution B in 4.5 min, then hold up until stop. Data were collected and analyzed by Analyst Software (AB Sciex).

### Xenograft Tumor Assay

According to the methods (Zhang et al., 2015), 3×10^6^ cancer cells were injected subcutaneously into the mammary fat pad near the fourth nipple of 6–8 week old BALB/c female mice. The mice were evenly distributed in three groups (8 mice/group), and four additional mice without any treatment (the mice had no injection of tumor cells and treatment with cantharidin, but raised at the same environment) were added as the control group. Except for the mice of the model and untreated groups with 5% glucose injection, other two groups were injected intraperitoneally with cantharidin (0.2 mg/kg, 0.5 mg/kg) every other day. At day 21 post-treatment, the mice were sacriﬁced and the liver and lung were collected for corresponding assays. The mice were purchased from Nanjing Biomedical Research Institute of Nanjing University, Nanjing, China. The study was authorized by the Animal Ethics Committee of Nanjing University of Chinese Medicine.

### Western Blot Analysis

This assay was performed according to the method as previously described (Chen et al., 2010). In brief, cancer cells were lysed in cold RIPA buffer after being washed with cold PBS. The lysates were sonicated and centrifuged by 14,000 rpm at 4°C; 20 μg protein was mixed with loading buffer; the mixture was loaded and separated on SDS-polyacrylamide gel. The target protein was then transferred from the Sodium dodecyl sulfate (SDS) gel to a polyvinylidene difluoride (PVDF) membrane. To eliminate the interference of non-specific proteins, the PVDF membrane was incubated with PBS buffer containing 5% non-fat milk. Finally, the membrane was respectively incubated with the corresponding primary antibodies and secondary antibodies. Immunoreactive bands were developed by enhanced chemiluminescence reagent. And the band images were taken by Bio-Rad XRS+ Gel System (Bio-Rad, Hercules, CA, USA). The following antibodies were used for this assay: PKM, PKM2, EGFR, GLUT1, PIN1, importin α5, MCT1, MCT4, lamin B1, and β-actin.

### Immunofluorescent Staining

Cells seeded on glass coverslips, which were laid in the bottom of a 6-well plate, were treated with cantharidin for 24 h, then fixed in 4% paraformaldehyde, permeabilized with 0.2% Triton X-100, and blocked in 1% BSA in turn. Next, the coverslips were incubated respectively with corresponding primary antibodies, secondary antibody, and Hoechst nuclear dye. Finally, the coverslips were observed by the Mantra Workstation with fluorescent microscopy and the data were analyzed using inForm 2.1.1 software (PerkinElmer, Waltham, MA, USA).

### Immunohistochemistry Staining

Serial sections (5 μm) were cut from formalin-fixed, paraffin-embedded lung, and liver tissues for IHC staining. After retrieving the antigen using citrate buffer (0.01 ml, pH 6.0), the sections were washed and incubated in endogenous peroxidase blockers for 10 min. The sections were then incubated overnight with antibodies at 4°C. After incubating with reaction enhancer before horseradish peroxidase-labeled anti-rabbit/mouse IgG antibody for 20 min at room temperature, the slides were prepared with DAB and analyzed by Mantra Workstation equipped with Olympus BX43 (×200); the positive staining intensities were determined by inForm 2.1.1 software and the mean optical density was calculated.

### Statistical Analysis

Each experiment was independently performed three times. The quantified results were determined using Student’s t test and one-way ANOVA with Dunnett test by GraphPad Prism software. P < 0.05 was considered to be statistically significant.

## Results

### Cantharidin Inhibits the Metastases of Breast Cancer *In Vitro* and *In Vivo*

**Figure 1:**
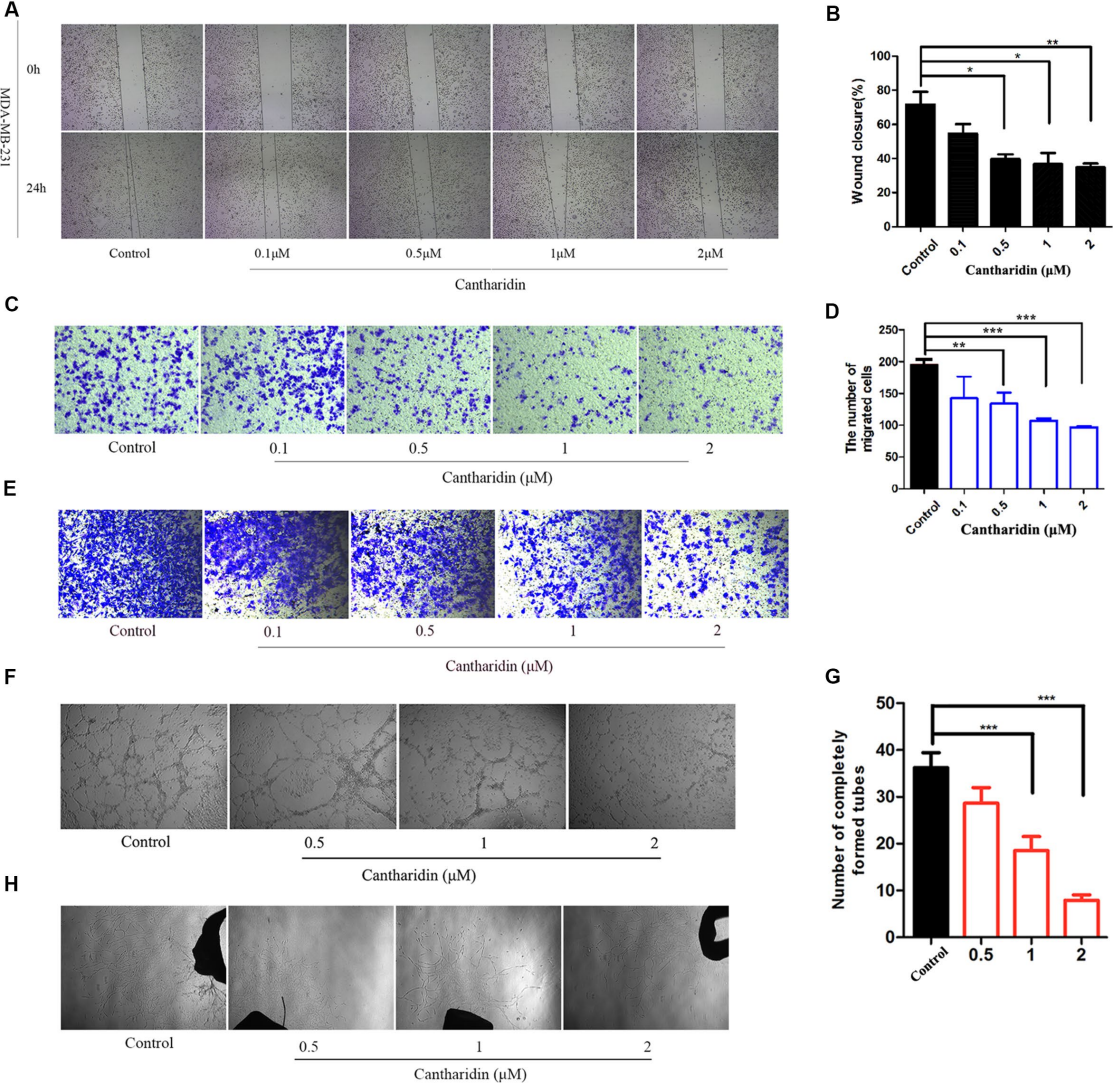
Cantharidin inhibits the metastasis of breast cancer *in vitro*. **(A)** Wound healing experiment in MDA-MB-231 cells. **(B)** Quantitation of wound closure rate. **(C–D)** Representative photos (200×) of transwell migration assay and quantitation analysis. **(E)** Representative photos (200×) of transwell invasion assay in MDA-MB-231 cells. **(F–G)** Representative images (40×) of tube formation from different treated groups. The number of completely formed tubes was quantitated. **(H)** Rat aortic ring assay with different treatment. Versus control, n = 3. *P < 0.05, **P < 0.01, ***P < 0.001.

Metastasis was considered a complicated process involving migration, infiltration, hematogenous and lymphatic metastasis, extravasation, and colonization of the secondary sites (Hanahan and Weinberg, 2011). This study demonstrated that cantharidin can block the multimetastatic processes. As shown in **Figure 1**, cantharidin significantly blocks the horizontal (**Figure 1A and B**) and vertical migration (**Figure 1C and D**) in MDA-MB-231 cells in a concentration-dependent manner. Meanwhile, cantharidin also decreases the number of invasive breast cancer cells (**Figure 1E**). Upon tube formation and rat aortic ring assay, we discovered that cantharidin can decrease the number of completely formed tubes (**Figure 1F and G**) and reduce the density and length of vascular sprouting (**Figure 1H**), indicating that angiogenesis can also be inhibited. The effective concentrations of cantharidin are by far less than its IC_50_ in MDA-MB-231 cells (**Figure S1**), further highlighting its anti-metastatic activity while avoiding its cytotoxicity.

**Figure 2:**
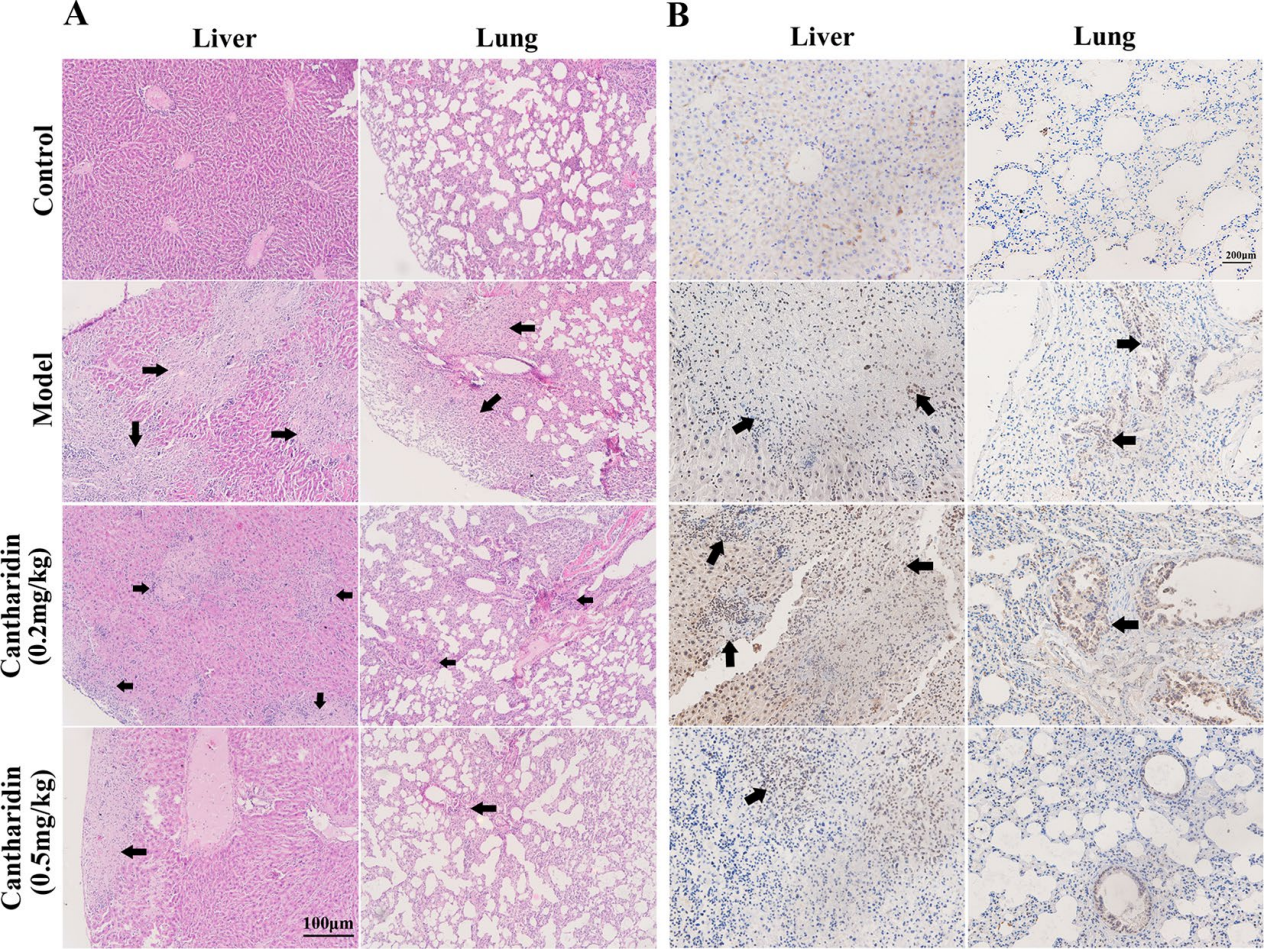
Cantharidin inhibits the metastasis of breast cancer *in vivo*. MDA-MB-231 cells were transplanted under the fourth mammary nipple. After 21-day treatment of cantharidin (0.2 and 0.5 mg/kg), the liver and lung tissues were collected for analysis of metastatic foci. **(A)** Representative HE staining images (100×). **(B)** Representative Ki-67 staining images (100×). Black arrows indicate the metastatic foci.

In orthotopic transplantation mice of breast cancer, the inhibitory effect of cantharidin on tumor metastasis was verified. Compared with the control group, the metastatic foci in the liver and lung tissues of the model group transplanted with MDA-MB-231 cells were identified clearly (**Figure 2A**), while 0.5 mg/kg cantharidin significantly reduces the metastatic foci and calcification area of the liver tissues (**Figure 2A**). Besides, the metastatic nodules could also be found through the ki-67 staining. As indicated in **Figure 2B**, the number and area of the region containing the metastatic cells in the liver and lung of the model group are more than those in the cantharidin group (0.5 mg/kg).

### Cantharidin Inhibits Aerobic Glycolysis

**Figure 3:**
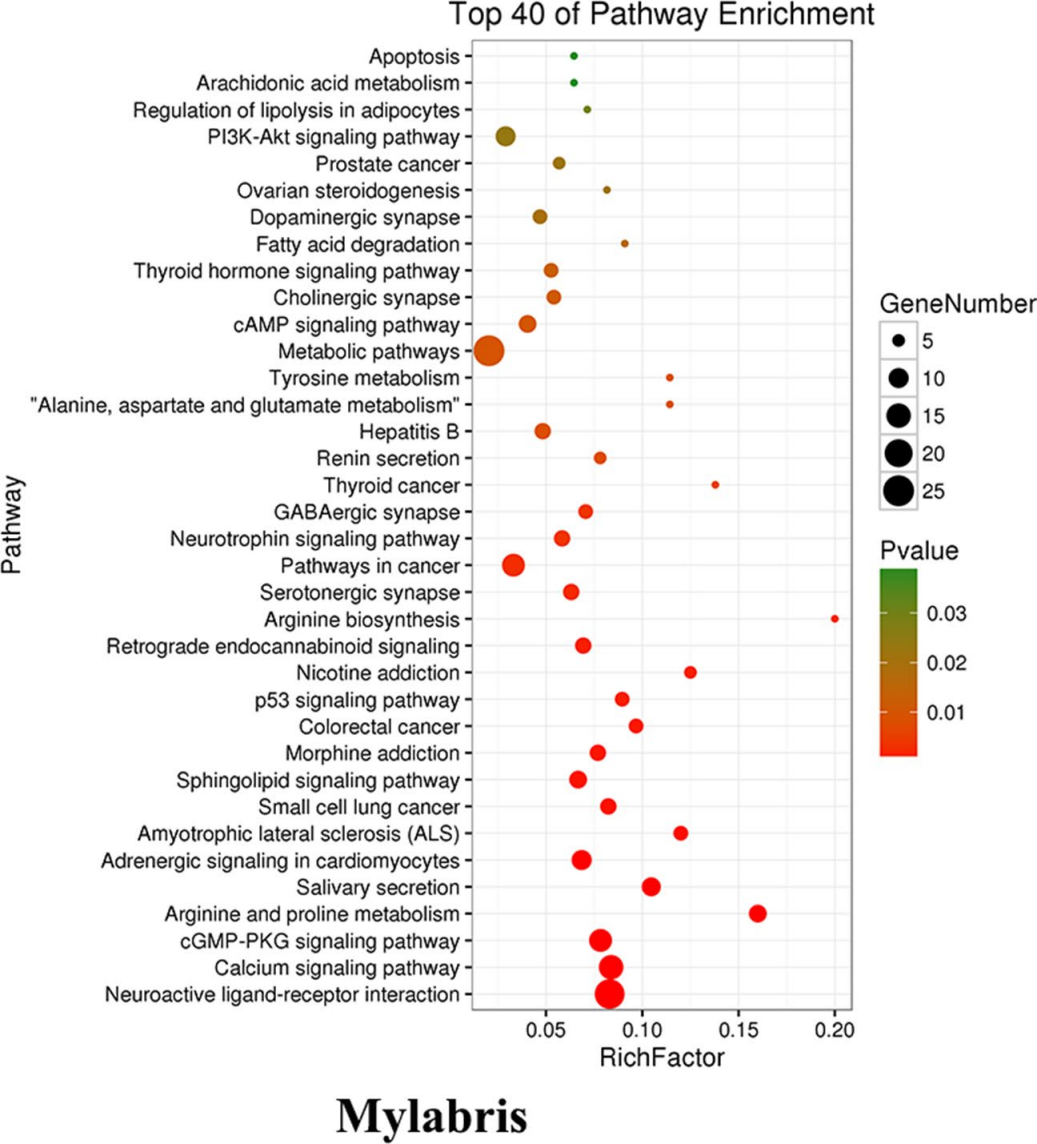
The enrichment analysis of putative targets for *Mylabris*.

**Figure 4:**
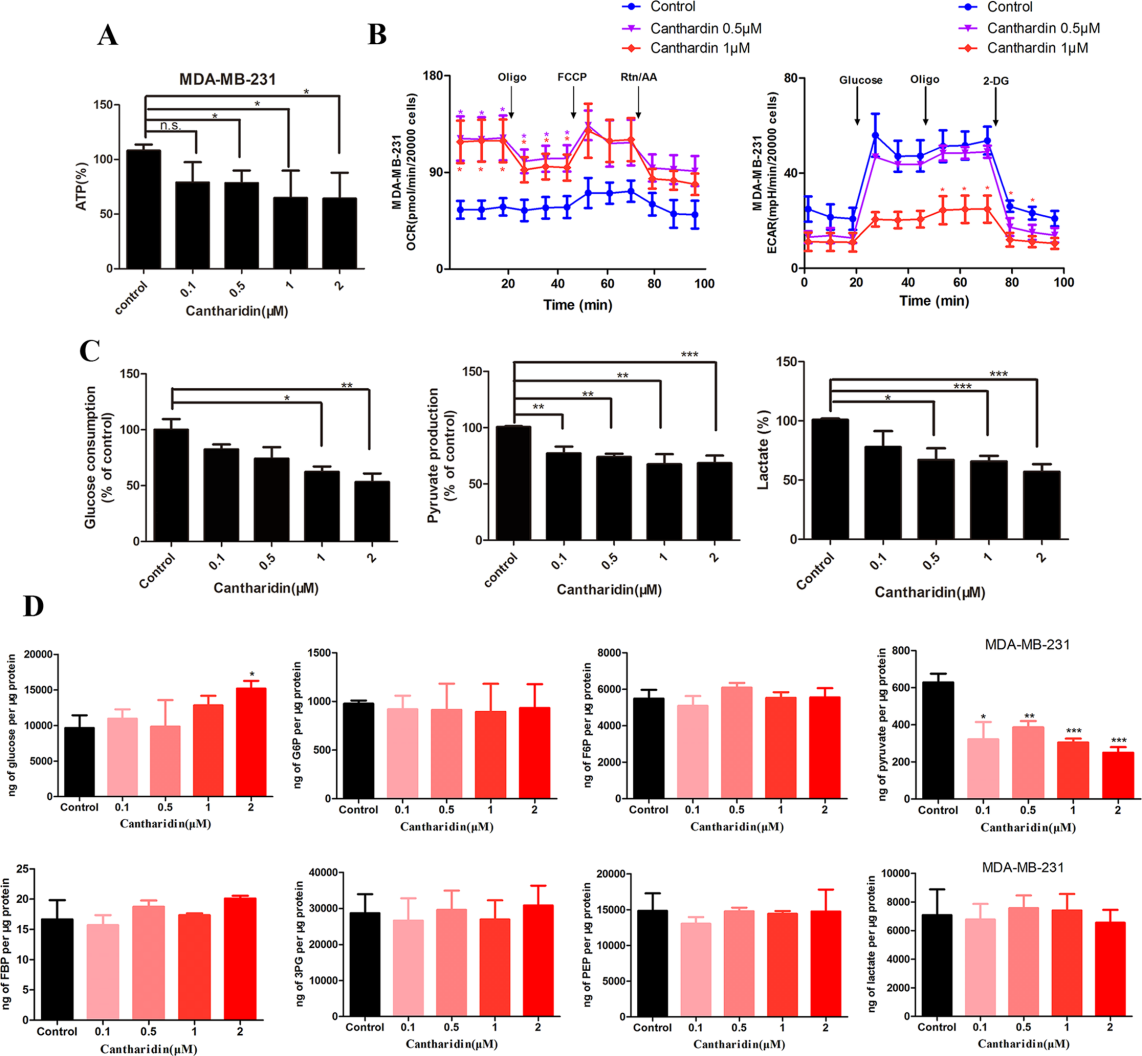
Cantharidin inhibits the aerobic glycolysis. MDA-MB-231 cells were treated with different concentrations of cantharidin for 24 h, then determined the following parameters using the corresponding reagent kits: **(A)** the ATP content, **(B)** the OCR and ECAR, **(C)** the level of glucose, pyruvate, and lactate. **(D)** The content of intracellular glycolytic metabolites was detected by HPLC-MS. Versus control, n = 3. *P < 0.05, **P < 0.01, ***P < 0.001.

As mentioned above, we firstly utilized the TCMSP website to search the active ingredients in *Mylabris*, and predicted their possible targets using the online program in the site of PharmMapper. Then we used an online database (QuickGO) and DAVID bioinformatic database for gene ontology (GO) analysis of the putative targets of *Mylabris* (Binns et al., 2009). The final results indicated that the enriched targets are focused on the metabolic pathways (**Figure 3**). According to the Warburg effect, the carcinoma prefers to metabolize by an aerobic glycolysis (Teoh and Lunt, 2018). Based on the aforementioned reports, the question has been raised whether cantharidin inhibition on metastasis was related to aerobic glycolysis. Thus, we tested the related parameters on glycolysis in breast cancer cells. Cantharidin no less than 0.5 μM can significantly decrease the ATP contents of breast cancer cells (**Figure 4A**). The data of ECAR and OCR measured by the XF24 extracellular flux analyzer indicated that cantharidin treatment caused a significant decrease in the ECAR and led to a notable increase in the OCR in MDA-MB-231 cells (**Figure 4B**), suggesting that cantharidin induces a metabolic transition from glycolysis to mitochondrial oxidative phosphorylation in breast cancer cells with high metastasis. To confirm our supposition, we analyzed three important metabolites in glycolysis process. The results indicated that the uptake of glucose and production of pyruvate, lactate are concentration-dependently inhibited by cantharidin (**Figure 4C**). Upon the intracellular metabolite analysis, only pyruvate is decreased (**Figure 4D**). And the similar results were attained in MCF-7 cells (**Figure S2**). Collectively, cantharidin inhibits cellular glycolytic metabolism in breast cancer cells.

### Cantharidin Inhibits Pyruvate Kinase Activity and PKM2 Nuclear Translocation

**Figure 5:**
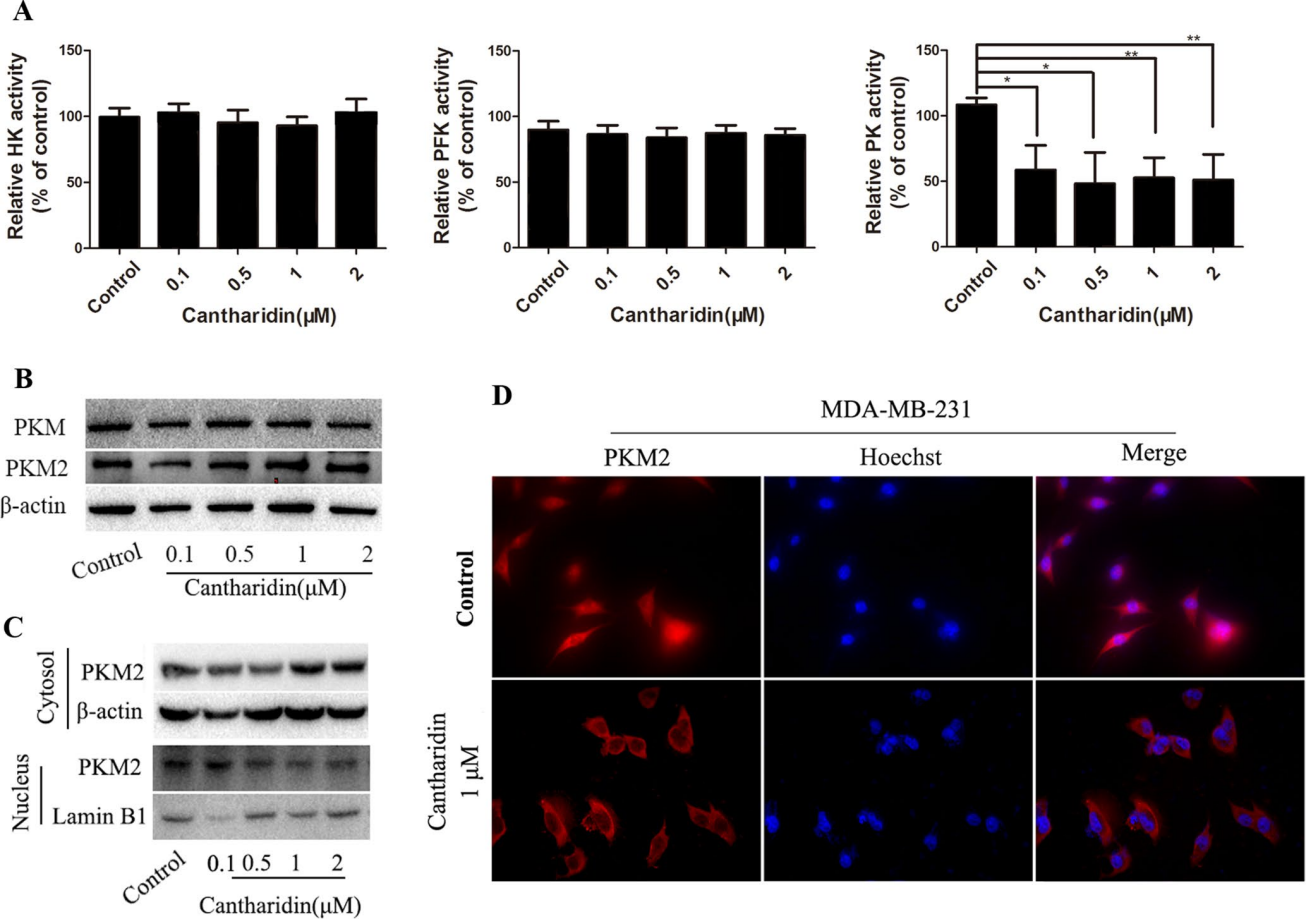
Cantharidin inhibits pyruvate kinase activity and PKM2 nuclear importation. MDA-MB-231 cells were treated with different concentrations of cantharidin for 24 h, then measured: **(A)** the activity of HK, PFK, and PK using the kinase reagent kit. **(B)** The protein levels of PKM, PKM2 by Western blotting. **(C)** The PKM2 expression in cytoplasm and nucleus by Western blotting. **(D)** The nuclear translocation of PKM2 by immunofluorescent analysis, and the representative images were indicated. Versus control, n = 3. *P < 0.05, **P < 0.01.

HK, PFK, and PK are three key kinases involved in aerobic glycolysis. HK catalyzes the essentially irreversible first step of the glycolytic pathway in which glucose is phosphorylated to glucose-6-phosphate (G6P) *via* phosphate transfer from ATP (Botzer et al., 2016). PK controls the last step of glycolysis, converting phosphoenolpyruvate (PEP) to pyruvate while phosphorylating ADP to ATP. All the glycolytic enzymes and transporters are overexpressed in cancer cells, with HK, PFK-1, and PKM2 showing the highest overexpression in human breast carcinomas (Moreno-Sánchez et al., 2012). In this study, only the activity of PK is obviously inhibited by cantharidin (**Figure 5A**); HK and PFK are not affected.

PK has four isoforms (M1, M2, L, and R), which express in different type cells and tissues. PKM2 expresses in most cells, especially in cancer cells, except adult muscle, brain, and liver, and is the predominant PK in cancer cells (Dong et al., 2016). However, cantharidin has no impact on the expression of PKM and PKM2 (**Figure 5B**). PKM2 normally presents in the cytoplasm in a homotetramer and acts as a metabolite kinase with high catalytic activity. A dimeric PKM2 form has been recently detected in nuclear extracts; the nuclear translocation stimulates its transcription to regulate the growth, survival, and metastasis of tumor cells (Gao et al., 2012; Filipp, 2013). Thus, PKM2 expression in the cytoplasm and nucleus was determined. Cantharidin significantly reduces the expression of PKM2 in the nucleus, while expression of cytosol PKM2 increases (**Figure 5C**). The inhibition of PKM2 nuclear translocation was further confirmed in the immunofluorescence test (**Figure 5D**). We also repeated the same determination in MCF-7 cells and got the similar data (**Figure S3**). Thus, we thought that PKM2 nuclear translocation mediates cantharidin inhibition of aerobic glycolysis.

### Cantharidin Inhibition on PKM2 Can Be Reversed by FBP or L-cysteine

**Figure 6:**
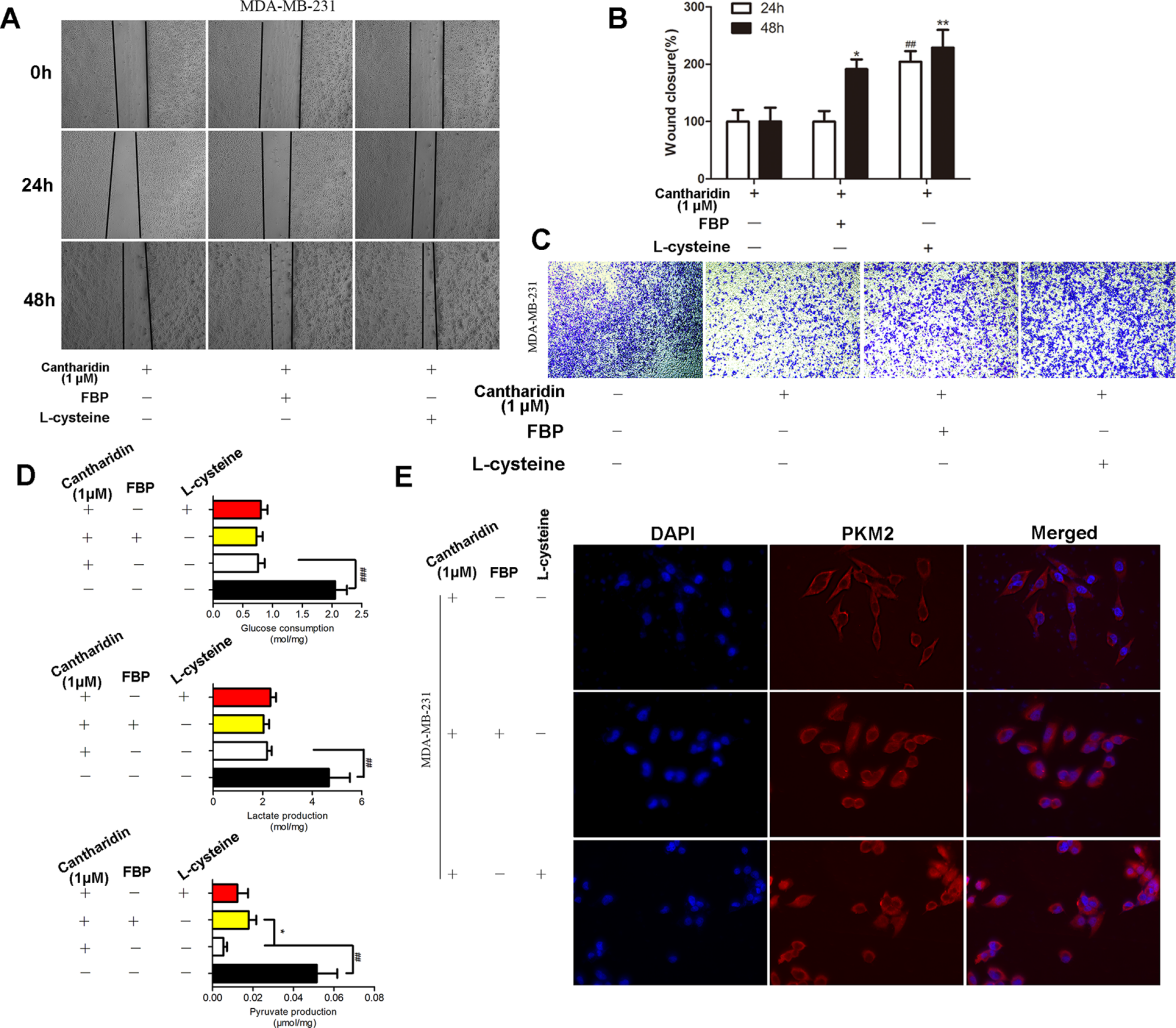
FBP and L-cysteine reverses the effect of cantharidin on MDA-MB-231 cells. MDA-MB-231 cells pretreated with FBP (100 µM) or L-cysteine (100 µM) were treated with 1 µM cantharidin for 24 h, then **(A, B)** cell migration, **(C)** invasion, and **(D)** metabolites of breast cancer cells were measured after 24-h treatment. **(E)** The immunofluorescent staining of PKM2. n = 3, *P < 0.05, **P < 0.01. ^#^ *P* < 0.05, ^##^ *P* < 0.01, ^###^ *P* < 0.001.

PKM2 fluctuates between two major states: a high active tetrameric form and a less active dimer, which associate with its catalytic activity and protein kinase, respectively. In the above study, we found that cantharidin obviously inhibited PK activity and the nuclear translocation of PKM2. When tetramer activator FBP or dimer activator L-cysteine were added, the inhibitory effect of cantharidin on migration and invasion was clearly reversed (**Figure 6A–6C**). Interestingly, both FBP and L-cysteine do not affect the inhibition of cantharidin on the uptake of glucose and production of lactate (**Figure 6D**), but FBP can reverse the inhibition of cantharidin on pyruvate production (**Figure 6D**). Most importantly, cantharidin inhibition on PKM2 translocation can be reversed by the FBP and L-cysteine as the PKM2 expression just stained in cytoplasm was determined in the whole cell (**Figure 6E**). Additionally, the similar data in MCF-7 were indicated in **Figure S4**. All above-mentioned further indicated that cantharidin could suppress PKM2 nuclear translocation, leading to inhibition of metastasis in the finally.

### Cantharidin Inhibits EGFR/GLUT1 Transduced Glycolytic Loop

**Figure 7:**
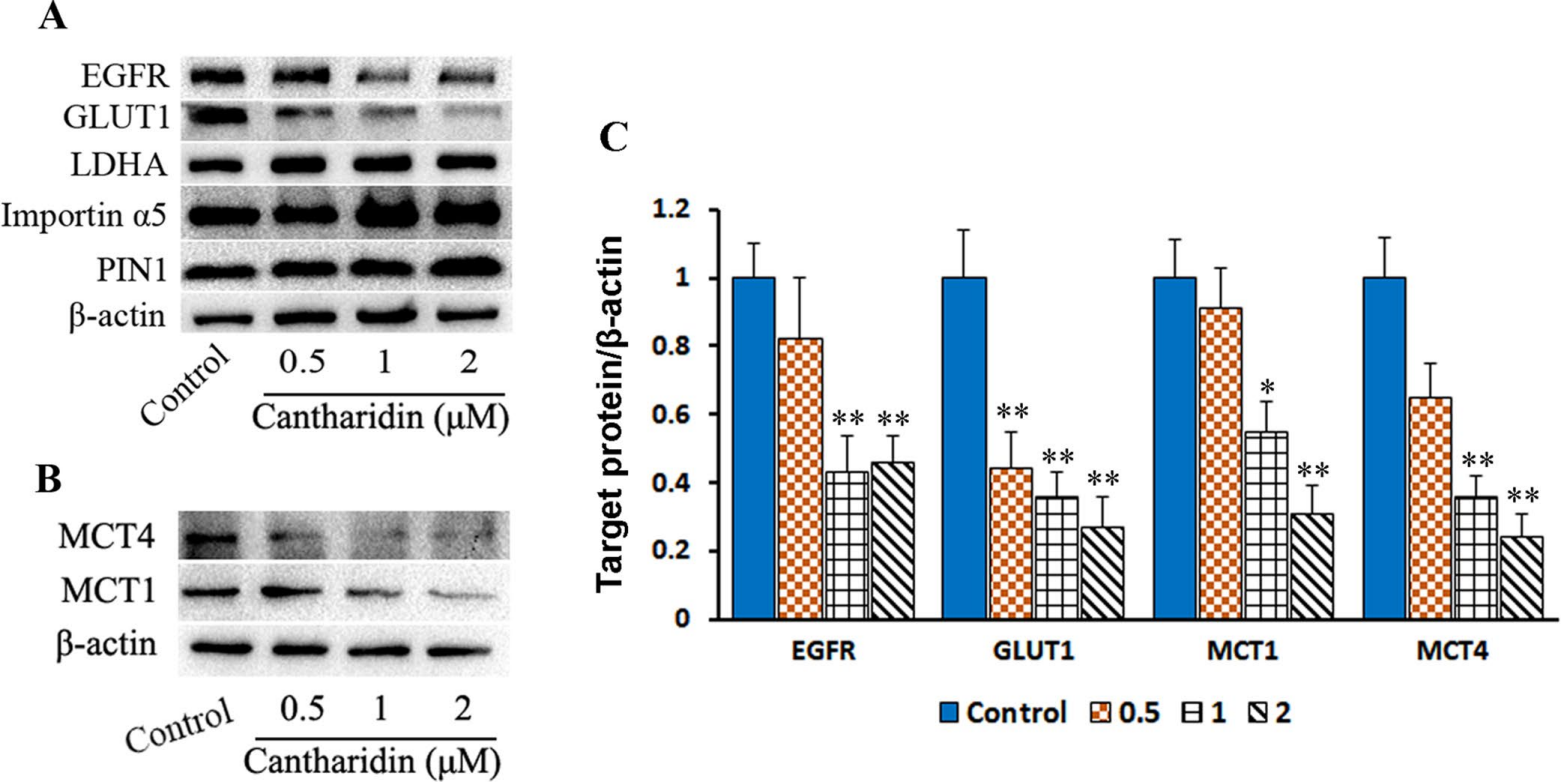
Cantharidin inhibits GLUT1 signal pathway. MDA-MB-231 cells were treated with different concentrations of cantharidin for 24 h, then executed: **(A)** the Western blot assay for determining the expression of EGFR, GLUT1, LDHA, importin α5, and PIN1. **(B)** The Western blot assay for the expression of MCT1 and MCT4. **(C)** The quantified analysis of the changed bands in **(A)** and **(B)**. Versus control, n = 3. *P < 0.005, **P < 0.01.

PKM2 nuclear translocation is also regulated by many signal pathways. One of the effectors pushing PKM2 entry into nuclear is due to activating EGFR and subsequently inducing PKM2 translocating into the nucleus, leading to the upregulation of GLUT1 and LDHA in a positive feedback loop (Yang et al., 2012). In this study, cantharidin does reduce the expression of GLUT1 but LDHA is unchanged in breast cancer cells (**Figure 7A and C** and **Figure S5A**). Furthermore, cantharidin obviously downregulates the expression of EGFR (**Figure 7A and C**), but not the PIN1 and importin α5 (**Figure 7A** and **Figure S5A**). Therefore, cantharidin inhibition of PKM2 nuclear translocation is independent on the PIN1 and importin α5, but related to EGFR and GLUT1. Taken together, the EGFR/GLUT1 mediated PKM2-controlled glycolytic loop is broken by cantharidin. In addition, we also found that cantharidin dose-dependently inhibits the expression of MCT4 and MCT1 (**Figure 7B and C** and **Figure S5B**), which are responsible for transporting the lactate out of the cells. This further reduces the level of lactate and facilitates inhibition of extracellular glycolytic process and metastasis.

## Discussion

PK plays a pivotal role in the final step of glycolysis and catalyzes PEP to form pyruvate by transferring a phosphate group. PKM2 is a key isoform of the PK that controls the cellular metabolism reprogramming. PKM2, regulated and transduced by many signal pathways, has tetrameric and dimeric forms, indicating high and low affinity to the substrate PEP, respectively (Mazurek et al., 2005). It was demonstrated that the dimeric and tetrameric PKM2 should be transferred mutually. And the allosteric effect of these two forms is directly activated by fructose-1,6-biphosphate (FBP), which is also a glycolytic intermediate product of PK. In cancer cells, dimeric PKM2 with less activity decreases the rate of glycolysis and increases the accumulation of FBP when it accumulated, while tetrameric PKM2 with high activity promoted by FBP increases the rate of glycolysis resulting in PKM2 dimerization (Dombrauckas et al., 2005). As a result, PKM2 was regarded as an attractive target for cancer treatment to interrupt the glycolysis. Furthermore, PKM2 is absolutely involved in the cancer metastasis though the related studies are limited at present. PKM2 signaling mediates the metastasis process, which can be promoted by a highly overexpressed lncRNA in colorectal cancer (Bian et al., 2018). Overexpression of PKM2 can stimulate the metastasis in hepatocellular carcinoma (Liu et al., 2015), or lymphatic metastasis in gastric cancer (Gao et al., 2015). In this study, cantharidin exhibits significant inhibition on the migration and invasion *in vitro* and breast cancer metastasis *in vivo*. ECAR-indicative glycolysis is inhibited by cantharidin as well as the ATP production and extracellular lactate, plus with that dimeric PKM2 transformation to tetramer is also blocked, suggesting cantharidin’s interruption on PKM2 nuclear translocation mediates its inhibition of breast cancer metastasis. Of course, the reversed effect of FBP and L-cysteine on cantharidin’s effects further supports the hypothesis.

**Figure 8:**
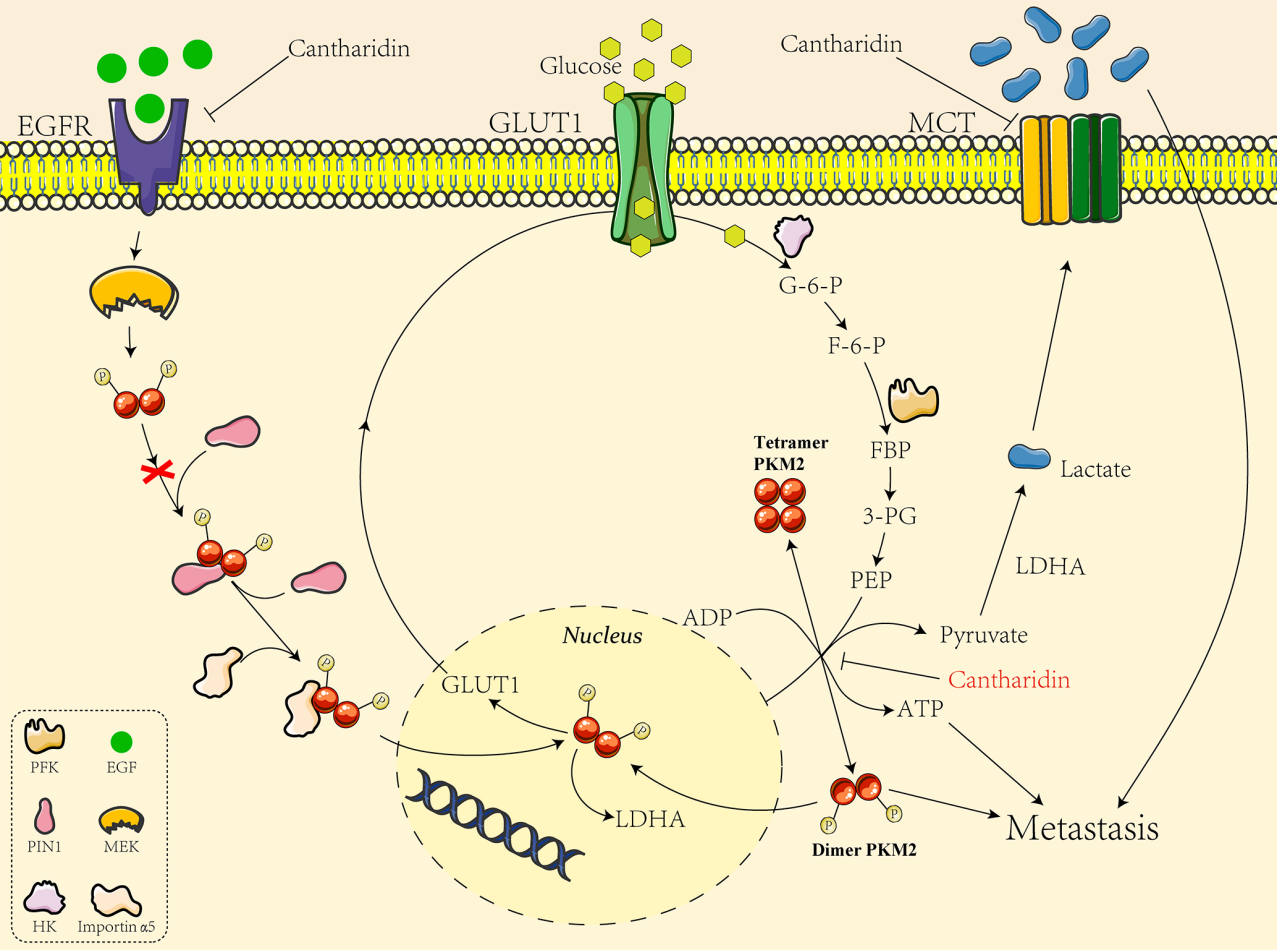
The mechanistic diagram of cantharidin inhibition of GLUT1-mediated PKM2 leading to metastasis blocked.

We also analyzed the mechanism underlying cantharidin’s effect on influencing PKM2 into the nucleus. Yang et al. (2011) discovered EGFR activation induced translocation of PKM2 into the nucleus, where K433 of PKM2 binds to c-Src-phosphorylated Y333 of β-catenin regulating cell proliferation and tumorigenesis. They later reported the specific mechanism of EGFR inducing PKM2 into the nucleus. EGFR-activated ERK2 phosphorylates PKM2 at Ser37, which recruits PIN1 for cis-trans isomerization of PKM2, promotes PKM2 binding to importin α5, and translocating to the nucleus subsequently followed with enhancement of transcription and expression of GLUT1 and LDHA (Yang et al., 2012). The glucose transported into cells by GLUT1 is glycolyzed by a series of enzymes including PK to form pyruvate, and the pyruvate is catalyzed to yield the lactate by LDHA. So the GLUT1/PKM2 mediated glycolytic metabolic loop is linked (**Figure 8**). Taken together, as indicated in **Figure 8**, GLUT1 controls the glucose transport, and dimer PKM2 is responsible for the glycolytic metabolism and also enters into the nucleus to promote the transcription of GLUT1 and LDHA, thus forming a glycolytic metabolic loop between GLUT1 and PKM2. Cantharidin inhibits PKM2 dimer transform from tetramer and entry into the nucleus, leading to the decrease of GLUT1 transcription and glucose consumption. Meanwhile, cantharidin only downregulates the expression of EGFR, but has no significant impact on the protein expression of PIN1 and importin α5, suggesting that EGFR may directly regulate GLUT1 or indirectly affect another pathway to mediate cantharidin’s inhibitory effect on the translocation of PKM2 into the nucleus. Furthermore, MCT1 and MCT4 responsible for exporting the intracellular lactate were downregulated by cantharidin, also matching the results that LDHA was unchanged but the extracellular lactate decreased. Totally, cantharidin inhibits multitarget proteins including EGFR, GLUT1, and PKM2 mediated glycolysis and MCTs, interfering the energy metabolism and finally leading to inhibition of breast cancer metastasis.

There are many kinds of mylabris-based and cantharidin-based pharmaceutical preparations on the Chinese market, such as mylabris capsules and disodium cantharidinate injection, all of which have been proved to show good anticancer effects (Dang and Zhu, 2013). A formulation specification for cantharidin patch (*Emplastrum cantharides*) had been included into the German Pharmacopeia (Thomas, 1926). Cantharidin has an excellent therapeutic effect on many kinds of tumors, but the clinical application of this vesicant compound was still limited by its insolubility, short half-life in circulation, and violent toxicity. Given its high toxicity, a lot of derivatives of cantharidin have been synthesized to avoid its toxicity but highlight its efficacy (Liu and Li, 1980; Zeng and Lu, 2006). Cantharidin already indicated anti-metastatic activity in low concentration of 1 μm far less than its IC_50_ cytotoxicity concentration. These implied that cantharidin particularly its derivatives would be potent anti-metastatic agents with highly applicable value in clinical setting.

## Data Availability Statement

All datasets generated for this study are included in the manuscript and/or the supplementary files.

## Ethics Statement

The animal study was authorized by the Animal Ethics Committee of Nanjing University of Chinese Medicine (ACU170402).

## Author Contributions

Conceptualization was done by WC and YL, methodology by YP, QZ, and SY, validation by MW and YL, formal analysis by ZW and SY, and investigation by YP, QZ, and WN. Resources were provided by AW. Data curation was performed by QJ, original draft preparation by YP, review and editing by WC, supervision by WC, and project administration by YL. WC and YL contributed to funding acquisition.

## Funding

The work was supported by National Natural Science Foundation of China (No. 81673648, 81673725, 81573859, 81673795) and Natural Science Foundation of Higher School of Jiangsu Province (17KJA360003).

## Conflict of Interest Statement

The authors declare that they have no conflict of interest.

## Supplementary Material

Figure S1 The cell viability of breast cancer cells treated with cantharidin. The cells with a density of 10^4^ cells/well were seeded into 96-well plates. After incubated overnight, the cells were exposed in the solution of cantharidin (0–80 µM) for 24 or 48 h. Then 20 µl MTS reagent was piped into the plate wells for chromogenic reaction. Finally, the optical density was measured at 570 nm by a BioTek microplate reader (BioTek Corporation, VT, USA) and the cell viability was calculated by the formula cell viability (%) = OD_treatments_/OD_control_ *100%.

Figure S2 Cantharidin inhibits the aerobic glycolysis. MCF-7 cells were treated with different concentrations of cantharidin for 24 h, then determined the following parameters using the corresponding reagent kits: **(A)** the ATP content, **(B)** the OCR and ECAR, **(C)** the level of glucose, pyruvate, and lactate. **(D)** The content of intracellular glycolytic metabolites was detected by HPLC-MS. Versus control, n = 3. **P*< 0.05, ***P*< 0.01, ****P*< 0.001.

Figure S3 Cantharidin inhibits pyruvate kinase activity and PKM2 nuclear importation. MCF-7 cells were treated with different concentrations of cantharidin for 24 h, then measured: **(A)** the activity of HK, PFK, and PK using the kinase reagent kit. **(B)** The protein levels of PKM, PKM2 by Western blotting. **(C)** The PKM2 expression in cytoplasm and nucleus by Western blotting. **(D)** The nuclear translocation of PKM2 by immunofluorescent analysis, and the representative images were indicated. Versus control, n = 3. **P*< 0.05, ***P*< 0.01.

Figure S4 FBP and L-cysteine reverses the effect of cantharidin. MCF-7 cells pretreated with FBP (100 µM) or L-cysteine (100 µM) were treated with 1 µM cantharidin for 24 h, then **(A, B)** cell migration, **(C)** invasion, and **(D)** metabolites of breast cancer cells were measured after 24-h treatment. **(E)** The immunofluorescent staining of PKM2. n = 3, **P*< 0.05, ***P*< 0.01. ^#^ *P*< 0.05, ^##^ *P*< 0.01, ^###^ *P*< 0.001.

Figure S5 Cantharidin inhibits GLUT1 signal pathway. MCF-7 cells were treated with different concentrations of cantharidin for 24 h, then executed: **(A)** the Western blot assay for determining the expression of EGFR, GLUT1, LDHA, importin α5, and PIN1. **(B)** The Western blot assay for the expression of MCT1 and MCT4.
