# Supplementary figures and images for: Breaking Glucose Transporter 1/Pyruvate Kinase M2 Glycolytic Loop Is Required for Cantharidin Inhibition of Metastasis in Highly Metastatic Breast Cancer
Source: Front Pharmacol. 2019 May 24;10:590. doi: 10.3389/fphar.2019.00590 (PMC6544055; doi:10.3389/fphar.2019.00590)

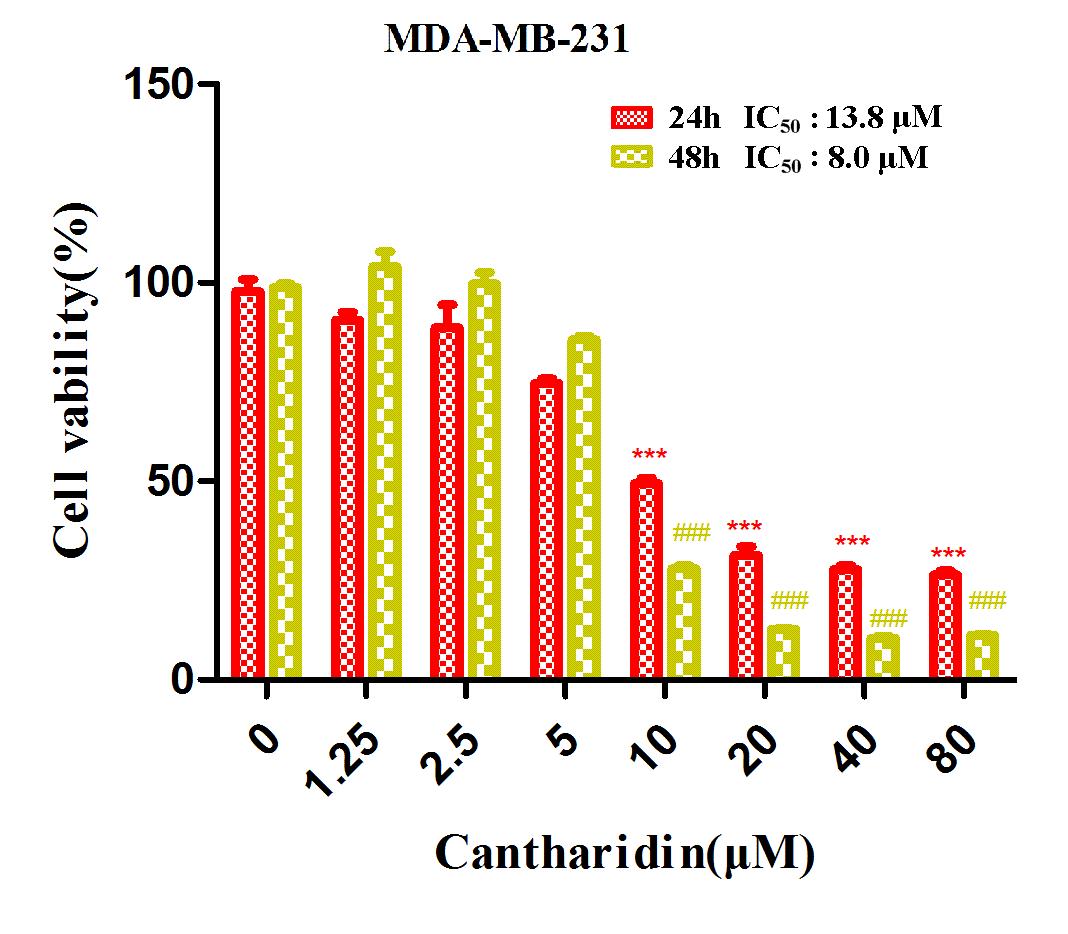

Supplement: Figure S1 — The cell viability of breast cancer cells treated with cantharidin. The cells with a density of 104 cells/well were seeded into 96-well plates. After incubated overnight, the cells were exposed in the solution of cantharidin (0–80 µM) for 24 or 48 h. Then 20 µl MTS reagent was piped into the plate wells for chromogenic reaction. Finally, the optical density was measured at 570 nm by a BioTek microplate reader (BioTek Corporation, VT, USA) and the cell viability was calculated by the formula cell viability (%) = ODtreatments/ODcontrol *100%. [file Image_1.tif]

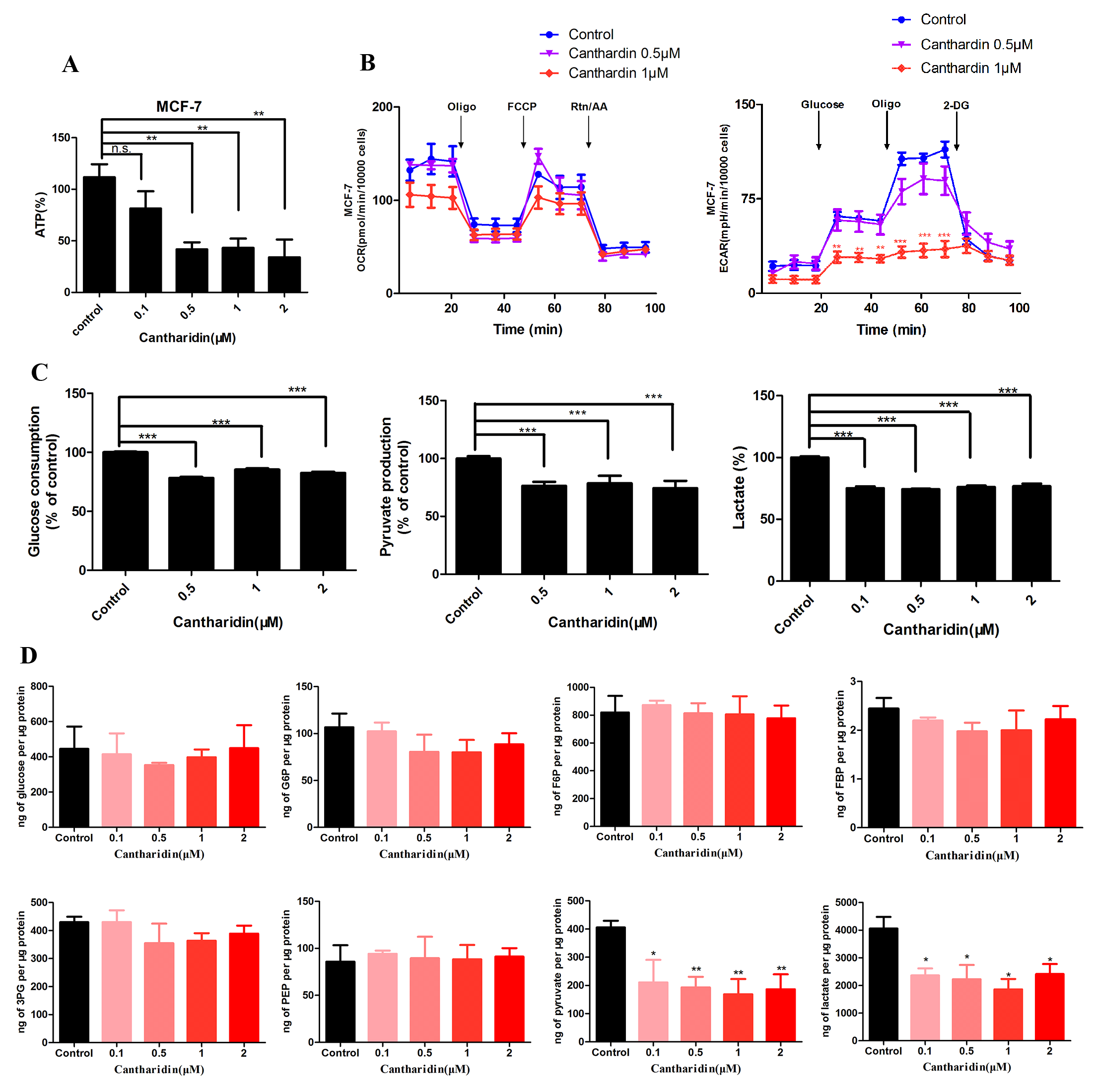

Supplement: Figure S2 — Cantharidin inhibits the aerobic glycolysis. MCF-7 cells were treated with different concentrations of cantharidin for 24 h, then determined the following parameters using the corresponding reagent kits: (A) the ATP content, (B) the OCR and ECAR, (C) the level of glucose, pyruvate, and lactate. (D) The content of intracellular glycolytic metabolites was detected by HPLC-MS. Versus control, n = 3. *P< 0.05, **P< 0.01, ***P< 0.001. [file Image_2.tif]

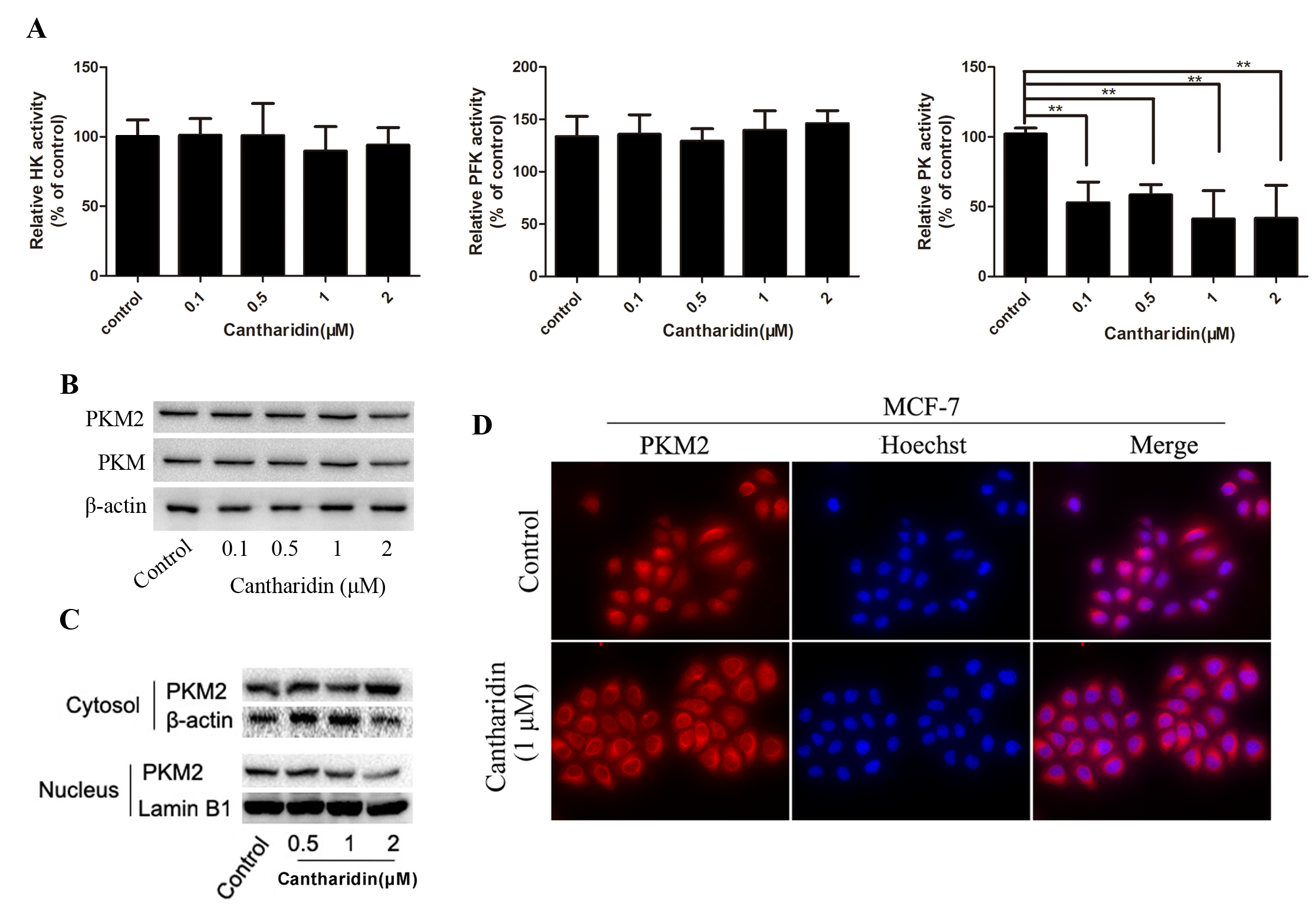

Supplement: Figure S3 — Cantharidin inhibits pyruvate kinase activity and PKM2 nuclear importation. MCF-7 cells were treated with different concentrations of cantharidin for 24 h, then measured: (A) the activity of HK, PFK, and PK using the kinase reagent kit. (B) The protein levels of PKM, PKM2 by Western blotting. (C) The PKM2 expression in cytoplasm and nucleus by Western blotting. (D) The nuclear translocation of PKM2 by immunofluorescent analysis, and the representative images were indicated. Versus control, n = 3. *P< 0.05, **P< 0.01. [file Image_3.tif]

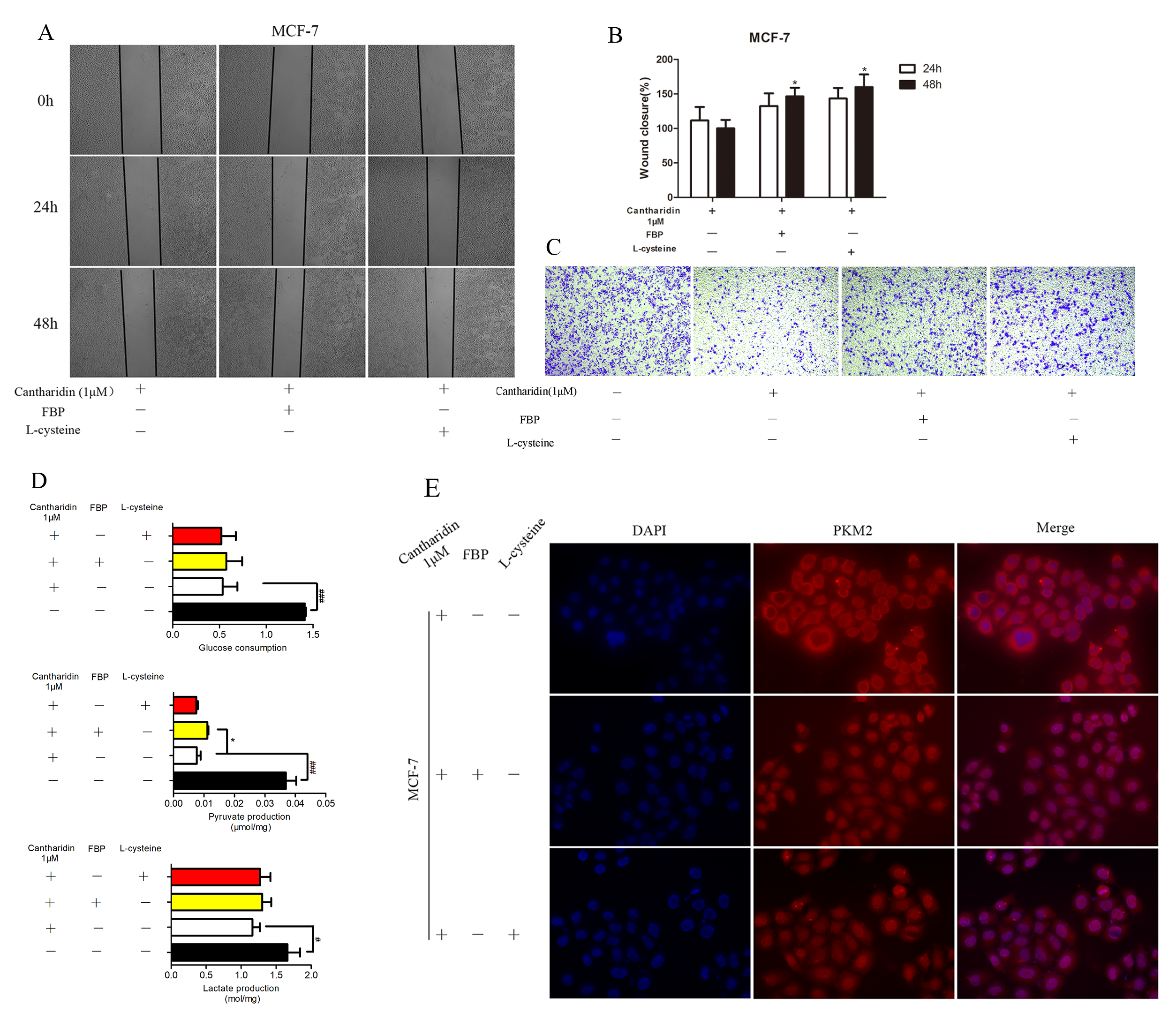

Supplement: Figure S4 — FBP and L-cysteine reverses the effect of cantharidin. MCF-7 cells pretreated with FBP (100 µM) or L-cysteine (100 µM) were treated with 1 µM cantharidin for 24 h, then (A, B) cell migration, (C) invasion, and (D) metabolites of breast cancer cells were measured after 24-h treatment. (E) The immunofluorescent staining of PKM2. n = 3, *P< 0.05, **P< 0.01. # P< 0.05, ## P< 0.01, ### P< 0.001. [file Image_4.tif]

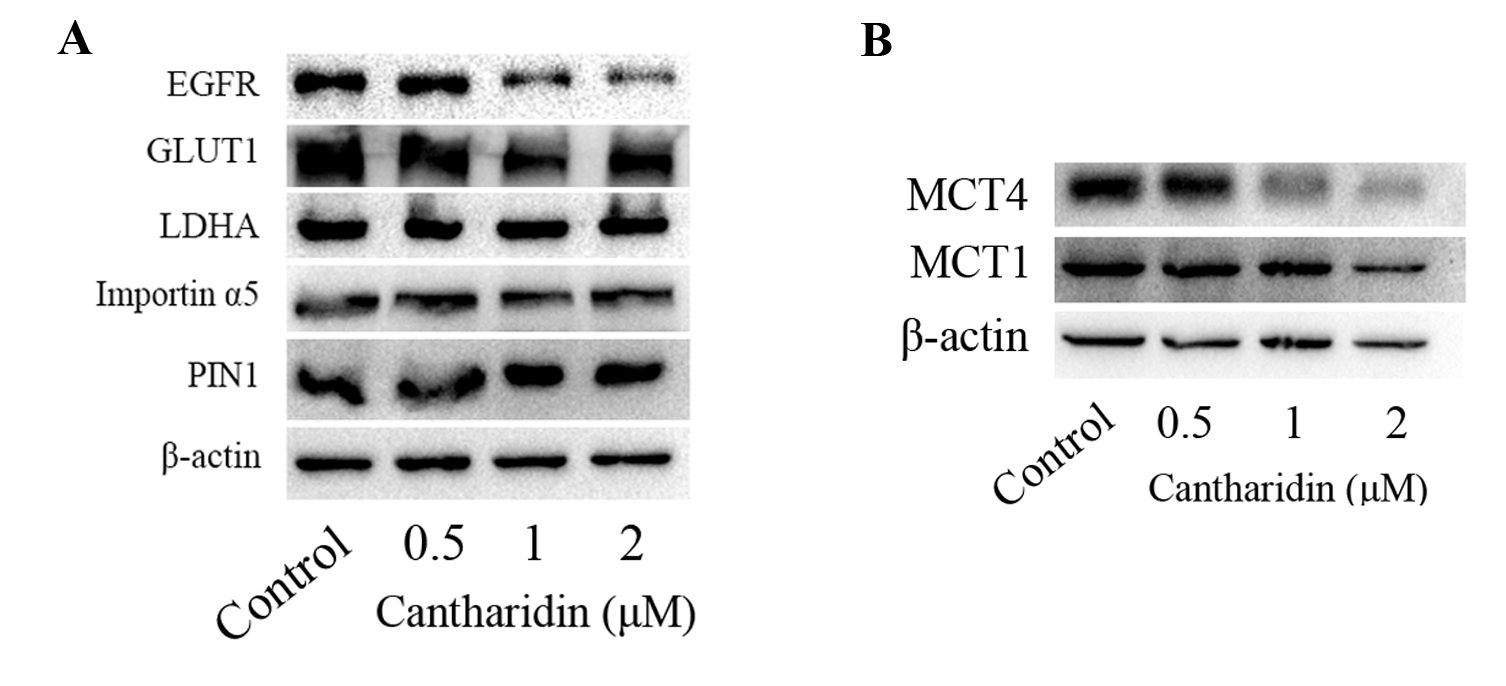

Supplement: Figure S5 — Cantharidin inhibits GLUT1 signal pathway. MCF-7 cells were treated with different concentrations of cantharidin for 24 h, then executed: (A) the Western blot assay for determining the expression of EGFR, GLUT1, LDHA, importin α5, and PIN1. (B) The Western blot assay for the expression of MCT1 and MCT4. [file Image_5.tif]
